# Cross-Cultural Review of Sexuality, Relationships, and Body Image after Burns: Analysis of the BSHS-B

**DOI:** 10.3390/ebj3010017

**Published:** 2022-02-24

**Authors:** Joseph S. Puthumana, Emily S. Ross, Patrick R. Keller, Carolyn S. Drogt, Kimberly H. Khoo, Eliana F. Duraes, Charles S. Hultman, Sheera F. Lerman

**Affiliations:** 1 Department of Plastic and Reconstructive Surgery, Johns Hopkins University School of Medicine, Baltimore, MD 21205, USA; eross26@gmail.com (E.S.R.); pkeller9@jh.edu (P.R.K.); cdrogt1@jhmi.edu (C.S.D.); kkhoo2@jhu.edu (K.H.K.); ferreie@ccf.org (E.F.D.); chultma1@jhmi.edu (C.S.H.); 2 Department of Psychiatry, Johns Hopkins University School of Medicine, Baltimore, MD 21205, USA; szohar1@jhmi.edu

**Keywords:** burns, sexuality, relationships, body image, BSHS-B, geographic, cultural

## Abstract

Burn survivors are at risk for dissatisfaction with body image, relationships, and sexuality due to disfiguring changes secondary to the injury. This review compares available global data on BSHS-B psychosocial scores. Twenty-four studies were included in the final analysis encompassing 14 countries; significant differences were found in scores across all BSHS-B psychological sub-sections of affect, body image, interpersonal relationships, and sexuality. On the whole, psychological well-being after burn injury was lower in Asian and South Asian countries compared to Europe or the United States. This study provides information for providers in burn centers caring for patients from a variety of cultural contexts and begins to steer initiatives to remedy psychological inequities in global burn care.

## 1. Introduction

Advancements in burn treatment have contributed to a significant increase in survival rates over the last 30 years [1]. As a result, goals of burn care have shifted beyond solely mortality to include functional and quality of life measures. Despite these advancements, burns remain some of the most traumatic injuries, with profound physical and psychological sequelae. Burn survivors face a unique set of psychological and psychosocial challenges that can compromise quality of life. The sudden and potentially disfiguring nature of the injury can lead to a discrepancy between ideal and perceived physical appearance, resulting in body image dissatisfaction [2]. Body image concerns may impact a patient’s readjustment and ability to cope following injury. Studies have found body dissatisfaction to be associated with greater depressive symptoms, increased social difficulties, lower levels of sexual satisfaction, and an overall lower quality of life [3,4,5]. Given these findings, it comes as no surprise that body image dissatisfaction, affect, sexuality, and interpersonal relationships have emerged as important psychosocial concerns in the rehabilitation process [6,7].

Body image dissatisfaction among burn survivors has been attributed to factors such as the severity of the injury and the importance of physical appearance [8]. Research has also shown that females report greater shame in post-injury body appearance compared to males [9]. Little is known, however, about how body image dissatisfaction and related factors, such as affect, sexuality, and interpersonal relationships, are impacted by a patient’s culture or geographic location. 

Body image dissatisfaction in the general population is often understood through the Tripartite Influence Model [10]. This model emphasizes the importance of culturally based appearance standards, proposing that three core sources (peers, parents, and media) influence body image attitudes, which are further mediated by the internalization of societal appearance standards and excessive appearance comparisons. For a long time, body dissatisfaction was limited to a Western phenomenon; however, studies have revealed that this is clearly not the case, and it is a prevalent issue across cultures [11]. Researchers have further examined the roles of ethnicity and culture in the development of body dissatisfaction in the general population. For instance, in comparison to white women, it was found that Asian women exhibited higher levels of body dissatisfaction [12]. Additionally, it was reported that in some Latina women, their ethnic identity may serve as a protective factor against negative body image and comparisons with Western beauty ideals [13]. These findings ultimately highlight the important role that culture may play in the development of body dissatisfaction, which needs to be better understood in the context of burn survivors.

Social and psychological outcomes in burn patients are commonly measured using the Burn Specific Health Scale (BSHS). Multiple forms of this instrument exist, including a 40-item BSHS-Brief version (BSHS-B) that was developed in response to the need for a shorter instrument to be used in the clinical setting [14]. The scale spans nine domains, including physical (hand function and simple abilities), burn-specific (heat sensitivity and treatment regimens), and social and emotional components (affect, work, sexuality, interpersonal relationships, and body image). The BSHS-B has undergone extensive psychometric testing over the last 30 years and has been translated, culturally adapted, and validated in multiple languages, including French [15], German [16], Persian [17], Arabic [18], Chinese [19], and Hindi [20], among others. In this review, we examine body image, affect, sexuality, and interpersonal relationship scores across a range of culturally adapted BSHS-B assessments. 

The aim of this systematic review was to assess BSHS-B scores in the psychosocial domains (body image, affect, sexuality, and interpersonal relationships) from a cross-cultural perspective. Considering the great diversity of burn patients in the United States, it is crucial to undertake this review to better understand how patients from other backgrounds will recover from burns in our burn centers, ultimately allowing us to provide more culturally competent psychosocial rehabilitation. This knowledge can additionally lend insight on what to expect as we build on global surgical burn infrastructure.

## 2. Materials and Methods

**Figure 1:**
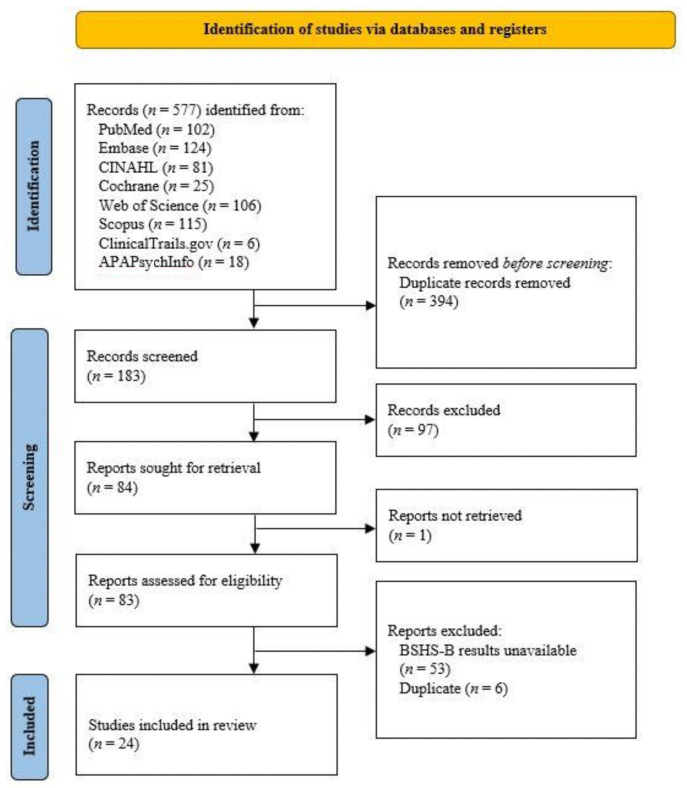
Selection of BSHS-B studies included in review.

A database search was conducted with “burn specific health scale-brief” OR “BSHS-B” in the title or abstract of articles published prior to 28 October 2021 across PubMed, CINAHL, Embase, Cochrane, Web of Science, Scopus, APAPsychoInfo, and clinicaltrials.gov. A total of 577 articles resulted, of which 369 were removed duplicates. Abstracts were screened by three authors (JP, PK, CD) for studies that investigated BSHS-B scores in a population rather than an ancillary endpoint in interventions. Each abstract was screened by two authors to ensure consistency. Two authors (JP, ER) screened studies in full-text review and excluded those without detailed BSHS-B data, including average scores broken down by component measures (e.g., affect, body image, interpersonal relationships, and sexuality) (Figure 1).

For each study, the following data items were collected: year of publication, country of publication, study population, number of participants, mean age and standard deviation, sex of participants, average TBSA and standard deviation, time elapsed between burn injury and BSHS-B administration, and BSHS-B component scores (on a 5-point scale ranging from 0–4). Higher BSHS-B psychosocial scores indicate higher health-related quality of life after burn injury. Absent data were noted for each study.

For statistical comparison, only studies with mean and standard deviation data available were included. In an attempt to better match cohorts, only studies outside the acute setting (>1 months) were included and divided into two groups: studies with patient cohorts suffering <20% TBSA burns and 20–40% TBSA burns, a cutoff previously used to signify a major burn [21]. Only two studies had >40% average TBSA burns, and these were not included in graphical representation or statistical comparison. For the geographic representation of data, “metameans” for countries with multiple studies were calculated [22,23].

One-way ANOVA was performed on collected summary data (number of patients, mean, and standard deviation) to compare BSHS-B scores across studies. All descriptive statistical analysis was performed using SPSS software (IBM Corp, Version 27, Armonk, NY, USA). A two-sided *p*-value ≤ 0.05 was considered significant.

## 3. Results

A total of 24 studies were included in the final analysis (Table 1). Studies were published between 2001 and 2021, with the majority published since 2011 (91.7%). The sizes of study samples ranged from 13 to 305, with an average *n* of 107.4 ± 76.3 patients. Follow-up, or time elapsed between burn injury and administration of BSHS-B, ranged from 0 days (day of discharge) to 170.4 ± 40.8 months. The age of participants was relatively consistent across studies, with most studies reporting an average age between 40 and 50 years (62.5%). There was a wider range of participant sex, ranging from 4% to 75.4% women.

**Figure 2:**
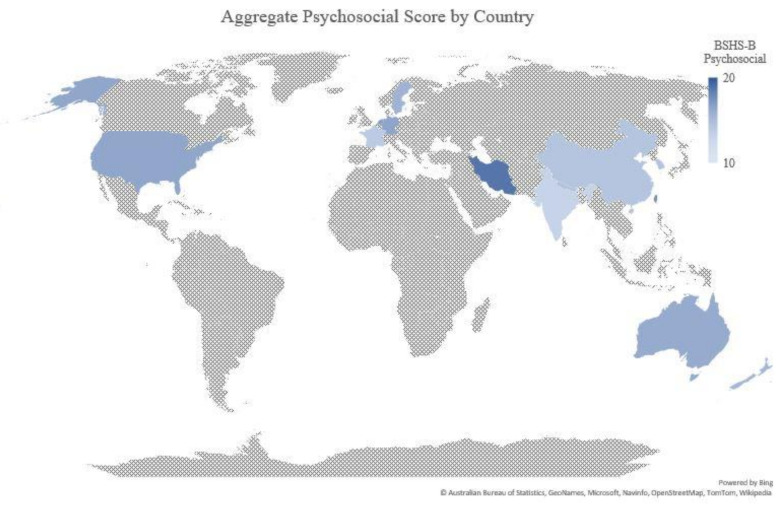
Map of aggregated mean by country of cumulative BSHS-B psychosocial score (sum of affect, body image, relationship, and sexuality scores).

In the 24 studies included, 14 countries were represented across four continents. The distribution of studies and associated aggregate mean psychosocial BSHS-B scores are presented in Figure 2. Categorized by World Bank classification, this review included lower-middle-income countries, upper-middle-inome countries, and high-income countries, but no low-income countries [45].

In studies with detailed affect, body image, relationship, and sexuality data, scores were statistically compared across studies and countries to better identify trends in psychosocial impact. To increase comparability, studies with patients that suffered smaller burns were separated from studies with larger burns.

**Figure 3:**
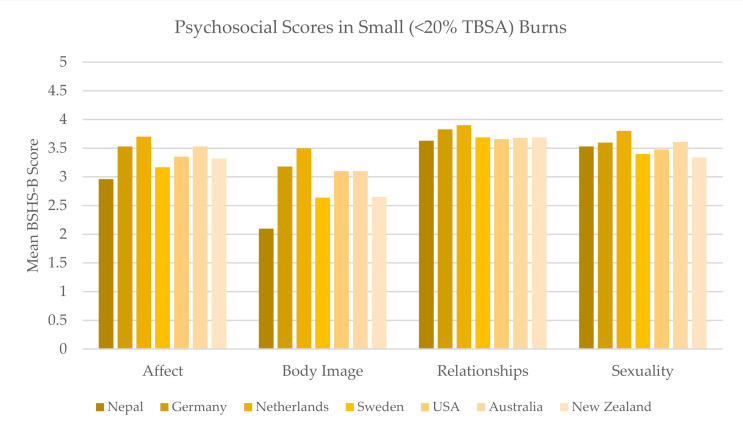
Mean BSHS-B score reported in studies with patient populations that suffered <20% TBSA burns on average.

The smaller burn cohort was comprised of seven studies across seven countries; there were significant differences across countries in affect (*p* < 0.001), body image (*p* < 0.001), relationships (*p* = 0.002), and sexuality (*p* = 0.004) (Figure 3). 

**Figure 4:**
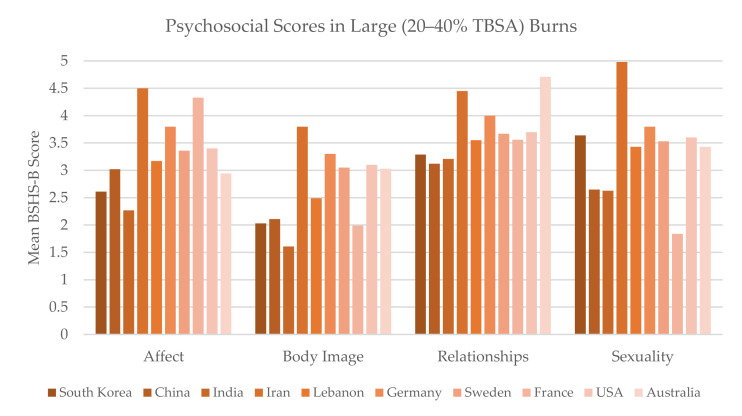
Mean BSHS-B score reported in studies with patient populations that suffered 20–40% TBSA burns on average.

Ten studies with large burn cohorts were compared. There were highly significant differences in all four measures: affect (*p* < 0.001), body image (*p* < 0.001), interpersonal relationships (*p* < 0.001), and sexuality (*p* < 0.001) (Figure 4).

## 4. Discussion

This multinational review included 24 studies across the geographic map and economic development spectrum in order to compile and quantitatively compare the psychosocial impacts of burn injuries. We found significant differences across studies in affect, body image, interpersonal relationships, and sexuality.

The studies compared in this review had several important commonalities: they were largely conducted within the last decade on a middle-aged patient population, with BSHS-B administered in the post-acute (>1 month) setting. Areas of discrepancy were primarily in TBSA and in the percentage of participants that were women, which varied drastically between studies and warrants a deeper future investigation given the reported negative impact of female sex on post-burn body image [9]. 

To minimize the contributing effects of burn size on psychosocial measures, we divided this study sample into two cohorts, <20% and 20–40%, a traditional cutoff for major burns for which altered physiology, hospital stay, and potential visibility of burns are exacerbated [21]. In the seven studies with patients that suffered smaller burns, interpersonal relationships and sexuality scores were found to be relatively consistent despite broad geographic variation in studies (Figure 3). This highlights the starker difference in body image, which is more negatively impacted by burn injuries in Nepal than in Germany, the Netherlands, Sweden, the United States, Australia, or New Zealand, six high-income countries that are likely able to provide burn patients with robust reconstructive services and psychological support. This is consistent with previously reported findings in the South Asian region, which showed that lack of access to primary burn care, reliance on mission trips for contracture management, and general cultural stigma negatively impact patients’ emotional and social well-being after burn injuries [46,47].

In the large burn cohort, there was highly significant variation across countries for all four measures: affect, body image, interpersonal relationships, and sexuality. In Figure 3, there is a general depression in scores in the Asian and Eastern Mediterranean region compared to Europe, the USA, and Australia, most prominently in affect and body image. Our findings may be read in the context of previous international work; one qualitative study identified that life for burn survivors is more difficult in societies with widespread “lookism”—in Korea, physical appearance is considered a skill, particularly for women, and visible burns have a profound impact on self-identity, well-being, and career opportunity [48,49]. In Chinese burn injuries, body and mind are theorized as a whole; physical defects are often considered related to moral defects, causing particular stigma [50]. One exception to this regional pattern is the Iranian study, which reported high BSHS-B scores despite previous work indicating societal stigma in Iran [51]. For future directions, further standardized research will be required to parse apart these geographic and cultural findings.

The findings of this review highlight the heterogeneity of psychosocial outcomes among burn survivors across the globe. Body image in particular showed significant geographic variation in both small and large burns. Various models have attempted to explain the development of body image dissatisfaction in the general population, including the well-established Tripartite Influence Model which focuses on the interplay between sociocultural and interpersonal factors [10]. In the context of this framework, our findings underscore the importance for providers to recognize how burn patients may experience differing culturally-influenced appearance ideals and differing degrees in the internalization of these ideals. Patients admitted to burn centers in an increasingly diversifying United States may come from a number of backgrounds; we hope that this review and its compilation of available data on body image, interpersonal relationships, and sexual impacts of burn injuries may help guide clinicians and psychological professionals as they treat these patients. In addition, as global burn care expands in the acute and reconstructive setting, this data may help guide attempts to remedy inequities in post-burn psychological care as well.

The most important limitation of this study is the limited sample size of 24 studies and the associated difficulty in making cross-national inferences in the psychosocial effects of burns. In particular, this study and the available research in BSHS-B have been concentrated in high-income countries and upper-middle income countries. Given the proliferating validation of the BSHS-B in other languages, from French to Arabic [15,16,17,18,19,20], and the ease of administration, we hope that additional data from low-income countries will emerge in the coming years. The generalization of our results should be performed with caution given that the studies compared in this review are inherently heterogeneous. However, we attempted to mitigate this by only quantitatively comparing studies with similar TBSA populations studied only in the post-acute setting. A centralized multi-institution, multi-national BSHS-B study will be required for a more accurate assessment of the cultural impacts of burn injury and to better account for the effects that variable follow-up may contribute to the findings presented here.

## 5. Conclusions

This study finds significant differences across 24 studies in 14 countries in post-burn psychosocial scores across affect, body image, interpersonal relationships, and sexuality. There are trends towards lower well-being scores, particularly in body image, in countries that more strongly stigmatize the prominent visual scarring sequelae of large burn injuries; providers should keep this cultural context in mind while treating patients from diverse backgrounds.

## Figures and Tables

**Table 1:** Studies with detailed BSHS-B scores, cohort size, time elapsed since burn, and demographic information included in final review (*n* = 24).

Author Name	Year	Country	Follow-Up	Age	Sex (% Women)	TBSA	*n*	Affect	Body Image	Interpersonal Relationships	Sexuality
Spronk et al. [24]	2021	Netherlands	67.2 ± 6 months	42.8 ± 13.5	37.1%	9.8 ± 12.7	155	3.7 ± 0.6	3.5 ± 0.8	3.9 ± 0.3	3.8 ± 0.5
Hsu et al. [25]	2021	Taiwan	36 months	21.35 ± 2.71	62.9%	61.12 ± 15.38	27	3.08 ± 0.54	2.11 ± 0.95	3.37 ± 0.75	3.45 ± 0.48
Tehranineshat et al. [26]	2020	Iran	80% in 6–12 months	34.48 ± 8.4	60.3%	36.1% ± 8.19	305	4.5 ± 0.27	3.8 ± 0.37	4.45 ± 0.79	4.98 ± 0.14
Shakya et al. [27]	2020	Nepal	39.9 ± 81.2 months	28.9 ± 10.6	57.7%	16.6 ± 8.3	111	2.96 ± 1.0	2.10 ± 1.3	3.63 ± 0.8	3.53 ± 0.9
Bourgi et al. [28]	2020	Lebanon	36.8 ± 16.1 months	44.6 ± 17.2	26.9%	24.5	130	3.17 ± 1.05	2.49 ± 1.41	3.55 ± 0.86	3.43 ± 0.81
Zhang et al. [29]	2019	China	116.72 days (15–427)	42.77 ± 13.82	36.4%	n/a	121	3.64 ± 0.62	3.08 ± 0.63	3.57 ± 0.49	3.81 ± 0.5
Gojowy et al. [30]	2019	Germany	170.4 ± 40.8 months	51 ± 17	26%	39 ± 17	42	3.8 ± 0.5	3.3 ± 0.8	4.0 ± 0.2	3.8 ± 0.6
Gandolfi et al. [31]	2018	France	>24 months	46.4 ± 15.9	34%	26.9 ± 15.9	53	4.33 ± 1.68	1.99 ± 1.18	3.56 ± 0.69	1.84 ± 0.76
Chin et al. [32]	2018	USA	120 ± 12 months	26 (12–44)	33.9%	22 (11–43)	221	3.6 (2.8–4.0)	3.0 (2.0–3.8)	4.0 (3.5–4.0)	4.0 (3.0–4.0)
Oh et al. [33]	2017	South Korea	0 (day of discharge)	44.52 ± 13.23	31%	14.94 ± 13.23	100	3.19 ± 0.90	2.72 ± 1.34	3.57 ± 0.71	3.28 ± 1.00
Berg et al. [34]	2017	Germany	11+ months	49.9 ± 15.2	35.6%	13.2 ± 11	141	3.53 ± 0.87	3.18 ± 0.98	3.83 ± 0.58	3.6
Wasiak et al. [35]	2017	Australia	12 months	M: 39.9 ± 17.6; F: 42.8 ± 13.6	75.4%	M: 18.5 (13–25); F: 14 (9–25)	114	3.53 ± 0.11	3.10 ± 0.14	3.68 ± 0.09	3.61 ± 0.10
Rothman et al. [36]	2016	USA	4.25 ± 6.72 months	41.32 ± 14.32	28.9%	87% with <30% TBSA	83	3.35 ± 0.78	3.1 ± 0.94	3.66 ± 0.55	3.48 ± 0.85
Ahuja et al. [37]	2016	India	10 months (8–12)	28 (23.75–32.25)	60%	30 (19.38–40.63)	60	2.22	3.92 ± 1.43	3.08 ± 1.29	2.29
Hwang et al. [38]	2016	Taiwan	18.6 ± 32.2 months	42.1 ± 13.3	36.1%	23.3 ± 25.4	108	3.85 (3.28–4)	4 (3.25–4)	4 (4–4)	4 (3.33–4)
Murphy et al. [39]	2015	USA	30–150 months	17.9 ± 1.7	44%	49.6 ± 12.5	50	3.4 ± 0.1	3.1 ± 0.1	3.7 ± 0.1	3.6 ± 0.1
Mulay et al. [20]	2015	India	6–12 months	30.95 (20–55)	60%	39.75 (20–60)	20	2.27 ± 1.38	1.61 ± 1.28	3.21 ± 1.24	2.63 ± 1.32
Dowda & Li [40]	2014	Australia	12 months	43 (21–81)	4%	21 (10–68)	13	2.94 ± 0.55	3.03 ± 0.81	4.71 ± 0.43	3.43 ± 0.59
Xie et al. [41]	2012	China	24–48 months	42.6 ± 13.0	30%	83.5 ± 9.7	20	3.0 ± 1.2	1.4 ± 1.1	3.6 ± 1.0	2.7 ± 2.5
Roh et al. [42]	2012	South Korea	2 ± 2 months	38.4 ± 10.8	29.2%	25.9 ± 15.9	113	2.61 ± 1.20	2.03 ± 1.33	3.29 ± 0.85	3.64 ± 0.68
Ling-Juan et al. [19]	2012	China	37.1 ± 35.4 months	40.42 ± 13.16	22.6%	40.05 ± 27.35	208	3.02 ± 1.09	2.11 ± 1.38	3.12 ± 1.15	2.65 ± 0.90
Reeve et al. [43]	2011	New Zealand	61 months	43.2 ± 12.3	36%	>1/2 10–20%	50	3.32 ± 1.01	2.65 ± 1.41	3.69 ± 0.72	3.34 ± 1.78
Sgroi et al. [44]	2005	Sweden	42 ± 14 months	43.7 ± 17.2	26.2%	16.7 ± 14.3	84	3.17 ± 0.92	2.64 ± 0.17	3.69 ± 0.64	3.40 ± 0.90
Kildal et al. [14]	2001	Sweden	111.6 ± 57.6 months	46.1 ± 15.5	19.8%	23.1 ± 16.2	248	3.36 ± 0.77	3.05 ± 1.00	3.67 ± 0.68	3.53 ± 0.77

## Data Availability

All studies reviewed in this paper have been appropriately cited and are publicly accessible.
